# (*R*_P_)-5-Methyl-2-(propan-2-yl)cyclo­hexyl phen­yl{phen­yl[(1-phenyl­eth­yl)amino]­meth­yl}phosphinate

**DOI:** 10.1107/S1600536812028243

**Published:** 2012-07-04

**Authors:** Meng Yang, Yong-Ming Sun, Qing-Gao Hou, Chang-Qiu Zhao

**Affiliations:** a College of Chemistry and Chemical Engineering, Liaocheng University, Shandong 252059, People’s Republic of China

## Abstract

In the title compound, C_31_H_40_NO_2_P, the P atom has a distorted tetra­hedral stereochemistry [bond-angle range about P = 103.33 (6)–115.24 (15)°] and has *R*_P_ chirality, which was confirmed crystallographically. The dihedral angles between the P-bonded phenyl ring and the other two phenyl rings are 40.4 (3) and 12.2 (2)°. In the crystal, a C—H⋯O inter­action links mol­ecules into chains which extend along [100].

## Related literature

For general background on chiral phospho­rus compounds, see: Perlikowska *et al.* (2004 ▶). For the structures of similar compounds, see: Meng *et al.* (2010 ▶); Liu *et al.* (2011 ▶).
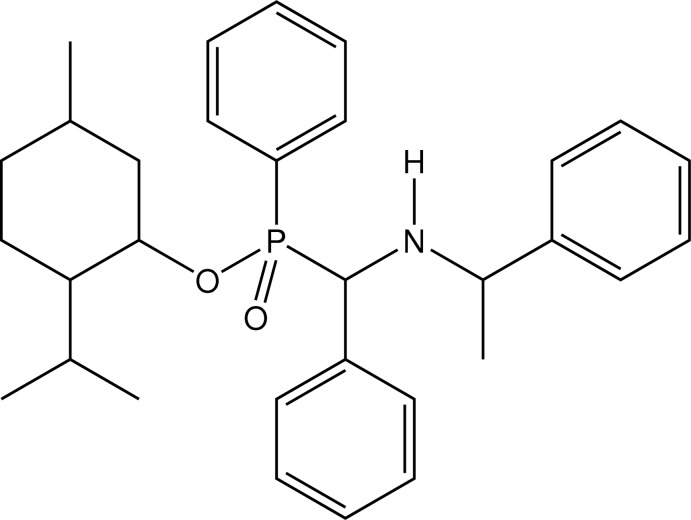


## Experimental

### 

#### Crystal data

C_31_H_40_NO_2_P
*M* *_r_* = 489.61
Orthorhombic, 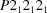

*a* = 5.8944 (4) Å
*b* = 11.4875 (11) Å
*c* = 43.795 (3) Å
*V* = 2965.5 (4) Å^3^
*Z* = 4
Mo *K*α radiation
μ = 0.12 mm^−1^
*T* = 298 K
0.48 × 0.18 × 0.11 mm

#### Data collection

Bruker SMART CCD area-detector diffractometer
Absorption correction: multi-scan (*SADABS*; Sheldrick, 1996 ▶) *T*_min_ = 0.945, *T*_max_ = 0.987
14971 measured reflections
5217 independent reflections
2787 reflections with *I* > 2σ(*I*)
*R*_int_ = 0.093

#### Refinement

*R*[*F* ^2^ > 2σ(*F* ^2^)] = 0.060
*wR*(*F* ^2^) = 0.133
*S* = 0.91
5217 reflections
320 parameters
H-atom parameters constrained
Δρ_max_ = 0.17 e Å^−3^
Δρ_min_ = −0.18 e Å^−3^
Absolute structure: Flack (1983 ▶), 2164 Friedel pairs
Flack parameter: −0.06 (15)

### 

Data collection: *SMART* (Bruker, 2007 ▶); cell refinement: *SMART*; data reduction: *SAINT* (Bruker, 2007 ▶); program(s) used to solve structure: *SHELXS97* (Sheldrick, 2008 ▶); program(s) used to refine structure: *SHELXL97* (Sheldrick, 2008 ▶); molecular graphics: *SHELXTL* (Sheldrick, 2008 ▶); software used to prepare material for publication: *SHELXTL*.

## Supplementary Material

Crystal structure: contains datablock(s) I, global. DOI: 10.1107/S1600536812028243/zs2207sup1.cif

Structure factors: contains datablock(s) I. DOI: 10.1107/S1600536812028243/zs2207Isup2.hkl

Supplementary material file. DOI: 10.1107/S1600536812028243/zs2207Isup3.cml

Additional supplementary materials:  crystallographic information; 3D view; checkCIF report

## Figures and Tables

**Table 1:** Hydrogen-bond geometry (Å, °)

*D*—H⋯*A*	*D*—H	H⋯*A*	*D*⋯*A*	*D*—H⋯*A*
C9—H9⋯O1^i^	0.98	2.50	3.454 (5)	164

Symmetry code: (i) 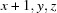.

**Fig. 1.**
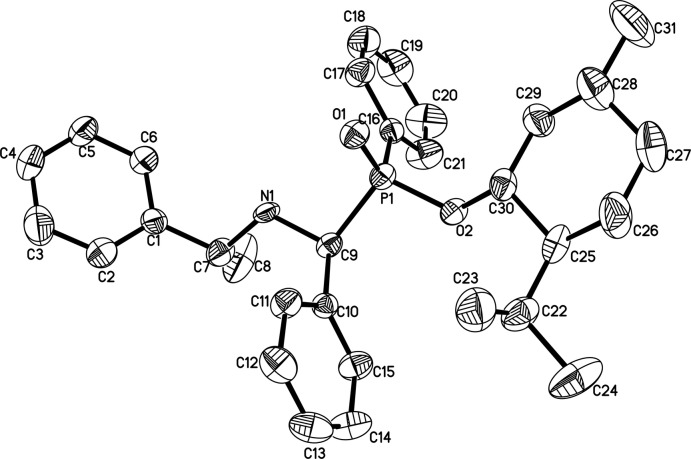
The molecular configuration and atom numbering scheme for the title compound showing 30% probability displacement ellipsoids. H atoms have been omitted.

**Fig. 2.**
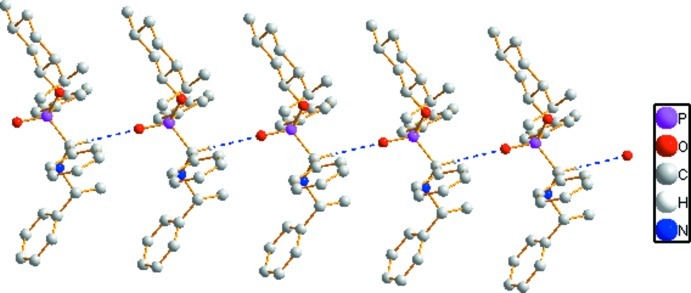
The one-dimensional hydrogen-bonded chain structure extending along a

## supplementary crystallographic information

### Comment

Chiral phosphorus compounds have been widely used in both chemistry and biology (Perlikowska *et al.*, 2004). The *P*-chiral title compound, C_31_H_40_NO_2_P, was synthesized using (*R*_P_)-*O*-(-)-menthyl phenylphosphinate in a reaction with the imine *N*-benzylidene-1-phenylethanamine and the structure is reported here. The structures of a number of the *O*-menthyl substituted phosphinates are known (Meng *et al.*, 2010; Liu *et al.*, 2011).

In this compound (Fig. 1) the P atom has a distorted tetrahedral stereochemistry [bond angle range about P1, 103.33 (6)–115.24 (15)°] and has *R*_P_ chirality, confirmed in the determination with the invoked absolute configuration for L-(-)-menthol [C1(*R*), C2(*S*), C5(*R*)] (equivalent to C30, C25, C28 respectively using the present atom numbering scheme). This also confirmed the configuration at the other two chiral centres as C7(*R*) and C9(*S*). The dihedral angles between the P-bonded phenyl ring (C16–C21) and the other two phenyl rings [C1–C6 and C10–C15] are 40.4 (3) and 12.2 (2)°, respectively.

In the crystal structure, there are no reasonable acceptors for the H-atom on N1 and only a single weak intermolecular methine C9—H···O(phosphinate) hydrogen-bonding interaction (Table 1) linking the molecules into chains extending along the *a* axial direction (Fig. 2). There are also 73 Å^3^ potential solvent accessible voids present in the unit cell.

### Experimental

(*R*_P_)-*O*-Menthyl phenylphosphinate (280 mg, 1 mmol) was added to (*N*)-benzylidene-1-phenylethanamine (209 mg, 1 mmol) in a flask and the mixture was stirred for 3 h at 80 °C. After washing with petroleum ether, the resulting solid was dried and recrystallized from diethyl ether to afford the pure title product.

### Refinement

All H atoms were positioned with idealized geometry with C—H = 0.93–0.98 Å or N—H = 0.86 Å and with *U*_iso_(H) = 1.2*U*_eq_(N and C aromatic, methylene or methine) or 1.5*U*_eq_(C methyl). The absolute configuration for L-(-)-menthol [C1(*R*), C2(*S*), C5(*R*)] was invoked (equivalent to C30, C25, C28, respectively using the present naming scheme), giving the configuration for the other three chiral centres as P1(*R*), C7(*R*), C9(*S*). The Flack parameter was -0.06 (15) for 2164 Friedel pairs.

### Crystal data

**Table:**

C_31_H_40_NO_2_P	*F*(000) = 1056
*M**_r_* = 489.61	*D*_x_ = 1.097 Mg m^−^^3^
Orthorhombic, *P*2_1_2_1_2_1_	Mo *K*α radiation, λ = 0.71073 Å
Hall symbol: P 2ac 2ab	Cell parameters from 1847 reflections
*a* = 5.8944 (4) Å	θ = 2.6–26.2°
*b* = 11.4875 (11) Å	µ = 0.12 mm^−^^1^
*c* = 43.795 (3) Å	*T* = 298 K
*V* = 2965.5 (4) Å^3^	Needle, colorless
*Z* = 4	0.48 × 0.18 × 0.11 mm

### Data collection

**Table:**

Bruker SMART CCD area-detector diffractometer	5217 independent reflections
Radiation source: fine-focus sealed tube	2787 reflections with *I* > 2σ(*I*)
Graphite monochromator	*R*_int_ = 0.093
φ and ω scans	θ_max_ = 25.0°, θ_min_ = 2.3°
Absorption correction: multi-scan (*SADABS*; Sheldrick, 1996)	*h* = −6→6
*T*_min_ = 0.945, *T*_max_ = 0.987	*k* = −12→13
14971 measured reflections	*l* = −45→52

### Refinement

**Table:**

Refinement on *F*^2^	Secondary atom site location: difference Fourier map
Least-squares matrix: full	Hydrogen site location: inferred from neighbouring sites
*R*[*F*^2^ > 2σ(*F*^2^)] = 0.060	H-atom parameters constrained
*wR*(*F*^2^) = 0.133	*w* = 1/[σ^2^(*F*_o_^2^) + (0.0472*P*)^2^] where *P* = (*F*_o_^2^ + 2*F*_c_^2^)/3
*S* = 0.91	(Δ/σ)_max_ = 0.001
5217 reflections	Δρ_max_ = 0.17 e Å^−^^3^
320 parameters	Δρ_min_ = −0.18 e Å^−^^3^
0 restraints	Absolute structure: Flack (1983), 2164 Friedel pairs
Primary atom site location: structure-invariant direct methods	Flack parameter: −0.06 (15)

### Special details

**Table:**

Geometry. All esds (except the esd in the dihedral angle between two l.s. planes) are estimated using the full covariance matrix. The cell esds are taken into account individually in the estimation of esds in distances, angles and torsion angles; correlations between esds in cell parameters are only used when they are defined by crystal symmetry. An approximate (isotropic) treatment of cell esds is used for estimating esds involving l.s. planes.
Refinement. Refinement of *F*^2^ against ALL reflections. The weighted *R*-factor *wR* and goodness of fit *S* are based on *F*^2^, conventional *R*-factors *R* are based on *F*, with *F* set to zero for negative *F*^2^. The threshold expression of *F*^2^ > σ(*F*^2^) is used only for calculating *R*-factors(gt) *etc*. and is not relevant to the choice of reflections for refinement. *R*-factors based on *F*^2^ are statistically about twice as large as those based on *F*, and *R*- factors based on ALL data will be even larger.

### Fractional atomic coordinates and isotropic or equivalent isotropic displacement parameters (Å^2^)

**Table:**

	*x*	*y*	*z*	*U*_iso_*/*U*_eq_	
P1	0.5520 (2)	0.76657 (8)	0.88670 (2)	0.0460 (3)	
O1	0.3082 (4)	0.8007 (2)	0.88595 (5)	0.0517 (7)	
O2	0.6330 (5)	0.6838 (2)	0.85959 (5)	0.0512 (8)	
C16	0.6336 (7)	0.6944 (3)	0.92173 (8)	0.0456 (10)	
C9	0.7490 (7)	0.8903 (3)	0.88450 (8)	0.0448 (10)	
H9	0.9020	0.8620	0.8892	0.054*	
N1	0.6732 (6)	0.9656 (3)	0.90980 (6)	0.0484 (9)	
H1	0.5492	0.9534	0.9196	0.058*	
C1	0.6882 (8)	1.1516 (4)	0.93517 (9)	0.0565 (12)	
C10	0.7547 (7)	0.9499 (3)	0.85307 (8)	0.0444 (10)	
C11	0.5780 (8)	1.0195 (3)	0.84330 (8)	0.0595 (12)	
H11	0.4505	1.0280	0.8556	0.071*	
C6	0.5898 (8)	1.1229 (4)	0.96267 (10)	0.0662 (13)	
H6	0.6112	1.0489	0.9708	0.079*	
C5	0.4582 (9)	1.2039 (4)	0.97855 (10)	0.0735 (14)	
H5	0.3930	1.1833	0.9971	0.088*	
C12	0.5873 (11)	1.0776 (4)	0.81516 (10)	0.0725 (14)	
H12	0.4659	1.1230	0.8087	0.087*	
C29	0.3980 (9)	0.5066 (4)	0.86151 (10)	0.0726 (14)	
H29A	0.3327	0.5312	0.8808	0.087*	
H29B	0.5328	0.4611	0.8659	0.087*	
C30	0.4635 (8)	0.6132 (3)	0.84289 (9)	0.0553 (11)	
H30	0.3274	0.6607	0.8397	0.066*	
C15	0.9441 (9)	0.9389 (3)	0.83448 (9)	0.0647 (12)	
H15	1.0650	0.8925	0.8407	0.078*	
C7	0.8250 (8)	1.0628 (4)	0.91695 (9)	0.0622 (13)	
H7	0.8729	1.0990	0.8978	0.075*	
C25	0.5629 (9)	0.5816 (4)	0.81157 (9)	0.0661 (13)	
H25	0.6976	0.5336	0.8153	0.079*	
C22	0.6407 (9)	0.6881 (5)	0.79321 (10)	0.0801 (16)	
H22	0.7477	0.7311	0.8061	0.096*	
C17	0.4904 (9)	0.7036 (3)	0.94717 (9)	0.0659 (14)	
H17	0.3523	0.7422	0.9455	0.079*	
C4	0.4242 (10)	1.3132 (4)	0.96705 (12)	0.0832 (15)	
H4	0.3334	1.3664	0.9774	0.100*	
C19	0.7526 (14)	0.5994 (5)	0.97769 (13)	0.099 (2)	
H19	0.7951	0.5692	0.9965	0.119*	
C13	0.7780 (11)	1.0666 (4)	0.79723 (11)	0.0797 (16)	
H13	0.7876	1.1063	0.7788	0.096*	
C21	0.8352 (10)	0.6366 (4)	0.92483 (11)	0.0832 (16)	
H21	0.9331	0.6305	0.9083	0.100*	
C23	0.4468 (12)	0.7729 (4)	0.78504 (10)	0.1058 (19)	
H23A	0.3358	0.7332	0.7729	0.159*	
H23B	0.5070	0.8375	0.7737	0.159*	
H23C	0.3770	0.8008	0.8034	0.159*	
C14	0.9541 (10)	0.9969 (4)	0.80666 (10)	0.0852 (15)	
H14	1.0813	0.9884	0.7943	0.102*	
C28	0.2269 (11)	0.4310 (4)	0.84434 (14)	0.103 (2)	
H28	0.0887	0.4768	0.8411	0.124*	
C18	0.5555 (13)	0.6546 (4)	0.97495 (10)	0.0934 (18)	
H18	0.4598	0.6606	0.9918	0.112*	
C26	0.3911 (11)	0.5050 (4)	0.79464 (11)	0.0977 (19)	
H26A	0.2565	0.5505	0.7901	0.117*	
H26B	0.4562	0.4799	0.7754	0.117*	
C27	0.3236 (12)	0.3980 (5)	0.81329 (13)	0.110 (2)	
H27A	0.2113	0.3536	0.8020	0.132*	
H27B	0.4556	0.3488	0.8162	0.132*	
C8	1.0347 (10)	1.0229 (5)	0.93405 (13)	0.123 (2)	
H8A	0.9902	0.9788	0.9517	0.184*	
H8B	1.1208	1.0896	0.9404	0.184*	
H8C	1.1260	0.9752	0.9209	0.184*	
C24	0.7718 (11)	0.6516 (6)	0.76396 (10)	0.131 (2)	
H24A	0.6666	0.6223	0.7491	0.196*	
H24B	0.8796	0.5919	0.7690	0.196*	
H24C	0.8500	0.7178	0.7557	0.196*	
C3	0.5261 (12)	1.3428 (4)	0.94010 (13)	0.107 (2)	
H3	0.5115	1.4178	0.9324	0.128*	
C2	0.6501 (10)	1.2608 (4)	0.92445 (10)	0.0914 (18)	
H2	0.7110	1.2812	0.9056	0.110*	
C31	0.1640 (14)	0.3211 (5)	0.86320 (14)	0.156 (3)	
H31A	0.2866	0.2661	0.8623	0.234*	
H31B	0.0294	0.2863	0.8549	0.234*	
H31C	0.1372	0.3429	0.8841	0.234*	
C20	0.8951 (11)	0.5864 (5)	0.95296 (14)	0.1029 (19)	
H20	1.0297	0.5447	0.9549	0.123*	

### Atomic displacement parameters (Å^2^)

**Table:**

	*U*^11^	*U*^22^	*U*^33^	*U*^12^	*U*^13^	*U*^23^
P1	0.0425 (7)	0.0519 (6)	0.0434 (5)	0.0031 (6)	−0.0007 (6)	−0.0033 (5)
O1	0.0402 (19)	0.0605 (16)	0.0545 (15)	0.0038 (13)	−0.0022 (15)	−0.0031 (14)
O2	0.049 (2)	0.0575 (15)	0.0469 (15)	0.0018 (14)	0.0050 (14)	−0.0043 (13)
C16	0.042 (3)	0.043 (2)	0.052 (2)	0.000 (2)	−0.001 (2)	−0.0006 (18)
C9	0.045 (3)	0.053 (2)	0.037 (2)	0.0080 (19)	0.002 (2)	0.0014 (19)
N1	0.049 (2)	0.061 (2)	0.0361 (17)	−0.0130 (19)	0.0178 (17)	−0.0120 (16)
C1	0.061 (3)	0.052 (3)	0.056 (3)	−0.019 (3)	0.007 (2)	−0.014 (2)
C10	0.043 (3)	0.047 (2)	0.044 (2)	−0.003 (2)	0.001 (2)	−0.0008 (18)
C11	0.062 (3)	0.066 (3)	0.051 (2)	0.013 (3)	0.004 (3)	0.006 (2)
C6	0.066 (4)	0.064 (3)	0.068 (3)	−0.002 (3)	0.010 (3)	−0.005 (2)
C5	0.077 (4)	0.078 (3)	0.065 (3)	0.004 (3)	0.016 (3)	−0.013 (3)
C12	0.077 (4)	0.071 (3)	0.070 (3)	0.002 (3)	−0.020 (3)	0.018 (2)
C29	0.083 (4)	0.066 (3)	0.069 (3)	−0.014 (3)	−0.001 (3)	−0.004 (2)
C30	0.041 (3)	0.061 (2)	0.064 (3)	−0.003 (2)	−0.008 (2)	−0.019 (2)
C15	0.058 (3)	0.083 (3)	0.053 (3)	0.006 (3)	0.001 (3)	0.007 (2)
C7	0.054 (3)	0.072 (3)	0.061 (3)	−0.011 (3)	0.003 (2)	−0.019 (2)
C25	0.067 (3)	0.073 (3)	0.059 (3)	0.021 (3)	−0.006 (3)	−0.023 (2)
C22	0.071 (4)	0.115 (4)	0.055 (3)	−0.018 (4)	0.005 (3)	−0.022 (3)
C17	0.077 (4)	0.069 (3)	0.052 (3)	0.011 (3)	0.008 (2)	0.007 (2)
C4	0.092 (4)	0.074 (3)	0.083 (4)	0.013 (3)	0.016 (3)	−0.022 (3)
C19	0.126 (6)	0.098 (4)	0.073 (4)	−0.001 (4)	−0.025 (4)	0.021 (3)
C13	0.085 (5)	0.097 (4)	0.057 (3)	−0.009 (4)	−0.011 (3)	0.034 (3)
C21	0.079 (4)	0.096 (3)	0.075 (3)	0.023 (3)	0.003 (3)	0.033 (3)
C23	0.145 (6)	0.093 (3)	0.080 (3)	0.007 (5)	0.001 (4)	0.001 (3)
C14	0.068 (4)	0.123 (4)	0.065 (3)	−0.002 (4)	0.015 (3)	0.018 (3)
C28	0.113 (5)	0.080 (4)	0.118 (5)	−0.025 (4)	−0.014 (4)	−0.009 (4)
C18	0.138 (6)	0.088 (4)	0.054 (3)	0.012 (4)	0.016 (4)	0.012 (3)
C26	0.123 (6)	0.085 (4)	0.085 (3)	−0.019 (4)	−0.021 (4)	−0.029 (3)
C27	0.135 (6)	0.081 (4)	0.115 (5)	−0.014 (4)	−0.025 (4)	−0.038 (4)
C8	0.062 (4)	0.165 (5)	0.142 (5)	0.019 (4)	−0.039 (4)	−0.091 (4)
C24	0.112 (6)	0.220 (7)	0.059 (3)	0.004 (5)	0.016 (3)	−0.011 (4)
C3	0.152 (7)	0.067 (3)	0.101 (4)	0.012 (4)	0.015 (5)	−0.007 (3)
C2	0.127 (5)	0.072 (3)	0.075 (3)	−0.003 (4)	0.038 (3)	−0.006 (3)
C31	0.197 (9)	0.107 (5)	0.164 (6)	−0.086 (5)	−0.008 (6)	0.002 (5)
C20	0.079 (5)	0.123 (4)	0.107 (4)	0.016 (4)	−0.021 (4)	0.041 (4)

### Geometric parameters (Å, º)

**Table:**

P1—O1	1.490 (3)	C22—C24	1.554 (6)
P1—O2	1.594 (3)	C22—H22	0.9800
P1—C16	1.809 (4)	C17—C18	1.395 (6)
P1—C9	1.837 (4)	C17—H17	0.9300
O2—C30	1.481 (4)	C4—C3	1.367 (6)
C16—C21	1.368 (6)	C4—H4	0.9300
C16—C17	1.401 (5)	C19—C18	1.329 (8)
C9—N1	1.475 (4)	C19—C20	1.379 (8)
C9—C10	1.538 (5)	C19—H19	0.9300
C9—H9	0.9800	C13—C14	1.375 (7)
N1—C7	1.464 (5)	C13—H13	0.9300
N1—H1	0.8600	C21—C20	1.405 (6)
C1—C2	1.358 (6)	C21—H21	0.9300
C1—C6	1.376 (5)	C23—H23A	0.9600
C1—C7	1.525 (6)	C23—H23B	0.9600
C10—C11	1.381 (5)	C23—H23C	0.9600
C10—C15	1.387 (6)	C14—H14	0.9300
C11—C12	1.402 (5)	C28—C27	1.522 (7)
C11—H11	0.9300	C28—C31	1.554 (7)
C6—C5	1.397 (6)	C28—H28	0.9800
C6—H6	0.9300	C18—H18	0.9300
C5—C4	1.367 (6)	C26—C27	1.528 (6)
C5—H5	0.9300	C26—H26A	0.9700
C12—C13	1.377 (7)	C26—H26B	0.9700
C12—H12	0.9300	C27—H27A	0.9700
C29—C30	1.521 (5)	C27—H27B	0.9700
C29—C28	1.529 (6)	C8—H8A	0.9600
C29—H29A	0.9700	C8—H8B	0.9600
C29—H29B	0.9700	C8—H8C	0.9600
C30—C25	1.535 (5)	C24—H24A	0.9600
C30—H30	0.9800	C24—H24B	0.9600
C15—C14	1.390 (6)	C24—H24C	0.9600
C15—H15	0.9300	C3—C2	1.375 (6)
C7—C8	1.516 (6)	C3—H3	0.9300
C7—H7	0.9800	C2—H2	0.9300
C25—C26	1.533 (6)	C31—H31A	0.9600
C25—C22	1.535 (6)	C31—H31B	0.9600
C25—H25	0.9800	C31—H31C	0.9600
C22—C23	1.544 (7)	C20—H20	0.9300
			
O1—P1—O2	115.43 (15)	C18—C17—H17	120.1
O1—P1—C16	113.32 (19)	C16—C17—H17	120.1
O2—P1—C16	106.18 (15)	C5—C4—C3	118.8 (5)
O1—P1—C9	113.88 (18)	C5—C4—H4	120.6
O2—P1—C9	103.45 (16)	C3—C4—H4	120.6
C16—P1—C9	103.35 (18)	C18—C19—C20	120.9 (5)
C30—O2—P1	119.5 (2)	C18—C19—H19	119.5
C21—C16—C17	118.7 (4)	C20—C19—H19	119.5
C21—C16—P1	122.5 (3)	C14—C13—C12	119.9 (4)
C17—C16—P1	118.6 (3)	C14—C13—H13	120.1
N1—C9—C10	114.7 (3)	C12—C13—H13	120.1
N1—C9—P1	102.9 (2)	C16—C21—C20	120.3 (5)
C10—C9—P1	113.9 (3)	C16—C21—H21	119.9
N1—C9—H9	108.4	C20—C21—H21	119.9
C10—C9—H9	108.4	C22—C23—H23A	109.5
P1—C9—H9	108.4	C22—C23—H23B	109.5
C7—N1—C9	115.0 (3)	H23A—C23—H23B	109.5
C7—N1—H1	122.5	C22—C23—H23C	109.5
C9—N1—H1	122.5	H23A—C23—H23C	109.5
C2—C1—C6	117.0 (4)	H23B—C23—H23C	109.5
C2—C1—C7	121.6 (4)	C13—C14—C15	120.7 (5)
C6—C1—C7	121.4 (4)	C13—C14—H14	119.7
C11—C10—C15	118.5 (4)	C15—C14—H14	119.7
C11—C10—C9	121.2 (4)	C27—C28—C29	109.5 (5)
C15—C10—C9	120.2 (4)	C27—C28—C31	111.2 (5)
C10—C11—C12	121.2 (5)	C29—C28—C31	110.9 (5)
C10—C11—H11	119.4	C27—C28—H28	108.4
C12—C11—H11	119.4	C29—C28—H28	108.4
C1—C6—C5	120.7 (4)	C31—C28—H28	108.4
C1—C6—H6	119.7	C19—C18—C17	120.8 (5)
C5—C6—H6	119.7	C19—C18—H18	119.6
C4—C5—C6	120.6 (4)	C17—C18—H18	119.6
C4—C5—H5	119.7	C27—C26—C25	112.0 (4)
C6—C5—H5	119.7	C27—C26—H26A	109.2
C13—C12—C11	119.3 (5)	C25—C26—H26A	109.2
C13—C12—H12	120.3	C27—C26—H26B	109.2
C11—C12—H12	120.3	C25—C26—H26B	109.2
C30—C29—C28	111.2 (4)	H26A—C26—H26B	107.9
C30—C29—H29A	109.4	C28—C27—C26	112.0 (4)
C28—C29—H29A	109.4	C28—C27—H27A	109.2
C30—C29—H29B	109.4	C26—C27—H27A	109.2
C28—C29—H29B	109.4	C28—C27—H27B	109.2
H29A—C29—H29B	108.0	C26—C27—H27B	109.2
O2—C30—C29	110.3 (3)	H27A—C27—H27B	107.9
O2—C30—C25	108.3 (4)	C7—C8—H8A	109.5
C29—C30—C25	112.7 (3)	C7—C8—H8B	109.5
O2—C30—H30	108.5	H8A—C8—H8B	109.5
C29—C30—H30	108.5	C7—C8—H8C	109.5
C25—C30—H30	108.5	H8A—C8—H8C	109.5
C10—C15—C14	120.3 (4)	H8B—C8—H8C	109.5
C10—C15—H15	119.8	C22—C24—H24A	109.5
C14—C15—H15	119.8	C22—C24—H24B	109.5
N1—C7—C8	112.0 (4)	H24A—C24—H24B	109.5
N1—C7—C1	107.4 (4)	C22—C24—H24C	109.5
C8—C7—C1	112.0 (4)	H24A—C24—H24C	109.5
N1—C7—H7	108.5	H24B—C24—H24C	109.5
C8—C7—H7	108.5	C4—C3—C2	119.6 (5)
C1—C7—H7	108.5	C4—C3—H3	120.2
C26—C25—C22	113.7 (4)	C2—C3—H3	120.2
C26—C25—C30	108.4 (4)	C1—C2—C3	123.2 (5)
C22—C25—C30	113.2 (3)	C1—C2—H2	118.4
C26—C25—H25	107.1	C3—C2—H2	118.4
C22—C25—H25	107.1	C28—C31—H31A	109.5
C30—C25—H25	107.1	C28—C31—H31B	109.5
C25—C22—C23	113.8 (4)	H31A—C31—H31B	109.5
C25—C22—C24	111.4 (4)	C28—C31—H31C	109.5
C23—C22—C24	110.3 (4)	H31A—C31—H31C	109.5
C25—C22—H22	107.0	H31B—C31—H31C	109.5
C23—C22—H22	107.0	C19—C20—C21	119.4 (6)
C24—C22—H22	107.0	C19—C20—H20	120.3
C18—C17—C16	119.8 (5)	C21—C20—H20	120.3
			
O1—P1—O2—C30	−21.9 (3)	C2—C1—C7—N1	−117.3 (5)
C16—P1—O2—C30	104.5 (3)	C6—C1—C7—N1	60.0 (5)
C9—P1—O2—C30	−147.0 (3)	C2—C1—C7—C8	119.4 (5)
O1—P1—C16—C21	167.2 (4)	C6—C1—C7—C8	−63.3 (6)
O2—P1—C16—C21	39.4 (4)	O2—C30—C25—C26	178.1 (3)
C9—P1—C16—C21	−69.1 (4)	C29—C30—C25—C26	55.8 (5)
O1—P1—C16—C17	−16.5 (4)	O2—C30—C25—C22	−54.8 (5)
O2—P1—C16—C17	−144.2 (3)	C29—C30—C25—C22	−177.1 (4)
C9—P1—C16—C17	107.2 (3)	C26—C25—C22—C23	61.4 (5)
O1—P1—C9—N1	54.3 (3)	C30—C25—C22—C23	−62.9 (5)
O2—P1—C9—N1	−179.6 (2)	C26—C25—C22—C24	−64.1 (6)
C16—P1—C9—N1	−69.1 (3)	C30—C25—C22—C24	171.6 (4)
O1—P1—C9—C10	−70.4 (3)	C21—C16—C17—C18	0.2 (6)
O2—P1—C9—C10	55.6 (3)	P1—C16—C17—C18	−176.2 (3)
C16—P1—C9—C10	166.2 (3)	C6—C5—C4—C3	−1.6 (8)
C10—C9—N1—C7	−65.7 (4)	C11—C12—C13—C14	1.6 (7)
P1—C9—N1—C7	170.0 (3)	C17—C16—C21—C20	0.8 (7)
N1—C9—C10—C11	−44.7 (5)	P1—C16—C21—C20	177.1 (4)
P1—C9—C10—C11	73.4 (4)	C12—C13—C14—C15	−1.3 (8)
N1—C9—C10—C15	132.2 (4)	C10—C15—C14—C13	0.5 (7)
P1—C9—C10—C15	−109.6 (4)	C30—C29—C28—C27	55.8 (6)
C15—C10—C11—C12	0.4 (6)	C30—C29—C28—C31	179.0 (5)
C9—C10—C11—C12	177.4 (4)	C20—C19—C18—C17	−1.8 (9)
C2—C1—C6—C5	−0.2 (7)	C16—C17—C18—C19	0.3 (8)
C7—C1—C6—C5	−177.7 (4)	C22—C25—C26—C27	178.2 (5)
C1—C6—C5—C4	0.1 (8)	C30—C25—C26—C27	−55.0 (6)
C10—C11—C12—C13	−1.2 (6)	C29—C28—C27—C26	−55.8 (7)
P1—O2—C30—C29	−78.2 (4)	C31—C28—C27—C26	−178.8 (5)
P1—O2—C30—C25	158.1 (3)	C25—C26—C27—C28	57.0 (7)
C28—C29—C30—O2	−178.7 (4)	C5—C4—C3—C2	3.3 (9)
C28—C29—C30—C25	−57.6 (5)	C6—C1—C2—C3	2.0 (8)
C11—C10—C15—C14	−0.1 (6)	C7—C1—C2—C3	179.4 (5)
C9—C10—C15—C14	−177.1 (4)	C4—C3—C2—C1	−3.6 (10)
C9—N1—C7—C8	−75.4 (4)	C18—C19—C20—C21	2.8 (9)
C9—N1—C7—C1	161.2 (3)	C16—C21—C20—C19	−2.3 (8)

### Hydrogen-bond geometry (Å, º)

**Table:**

*D*—H···*A*	*D*—H	H···*A*	*D*···*A*	*D*—H···*A*
C9—H9···O1^i^	0.98	2.50	3.454 (5)	164
C22—H22···O2	0.98	2.50	2.908 (5)	105
C30—H30···O1	0.98	2.59	3.006 (4)	106

Symmetry code: (i) *x*+1, *y*, *z*.
